# Cooper Pairs Distribution function for bcc Niobium under pressure from first-principles

**DOI:** 10.1038/s41598-021-87028-x

**Published:** 2021-04-07

**Authors:** G. I. González-Pedreros, J. A. Camargo-Martínez, F. Mesa

**Affiliations:** 1 Faculty of Natural Sciences, Universidad del Rosario, Carrera 24 # 63C-69, 111221 Bogotá, D.C. Colombia; 2 Grupo de Investigación en Ciencias Básicas, Aplicación E Innovación - CIBAIN, Unitrópico, Yopal, Colombia

**Keywords:** Condensed-matter physics, Superconducting properties and materials

## Abstract

In this paper, we report Cooper Pairs Distribution function ${D}_{cp}(\omega ,{T}_{c})$ for bcc Niobium under pressure. This function reveals information about the superconductor state through the determination of the spectral regions for Cooper-pairs formation. ${D}_{cp}(\omega ,{T}_{c})$ is built from the well-established Eliashberg spectral function and phonon density of states, calculated by first-principles. ${D}_{cp}(\omega ,{T}_{c})$ for Nb suggests that the low-frequency vibration region $\left(\omega <6 \,{\text{meV}}\right)$ is where Cooper-pairs are possible. From ${D}_{cp}(\omega ,{T}_{c})$, it is possible to obtain the ${N}_{cp}$ parameter, which is proportional to the total number of Cooper-Pairs formed at a temperature ${T}_{c}$. The ${N}_{cp}$ parameter allows an approach to the understanding of the Nb ${T}_{c}$ anomalies, measured around 5 and 50 GPa.

## Introduction

Niobium (Nb) is a conventional superconductor with a body-centered cubic (bcc) structure. It has a lattice constant of 6.24 Bohr^1^ and a superconducting critical temperature $\left({T}_{c}\right)$ of 9.25 K^2^, Nb is the element with the highest ${T}_{c}$. Critical temperature measurements under pressure ${T}_{c}(P)$ by Struzhkin et al*.*^3^ show discontinuities of ${T}_{c}$ around $4{-}5\,{\text{GPa}}$ and $50{-}60 \,{\text{GPa}}$, where ${T}_{c}$ increases by 0.7 K and decreases by about 1 K, respectively. These discontinuities are known as the Nb ${T}_{c}$ anomalies. The authors suggest that the first behavior is explained by stress-sensitive electronic topological transitions. The second one, from where ${T}_{c}$ drops continuously to 4.7 K, is related to the decrease in density of states at the Fermi level with increasing pressure.

Theoretical analysis of electronic band structure of Nb at $0{-}10\,{\text{GPa}}$ pressure interval does not show an appreciable variation. By contrast, the band structure behavior at $40{-}70 \,{\text{GPa}}$ could explain the origin of ${T}_{c}$ anomaly at this pressure range^4–6^. Wierzbowska et al*.*^5,6^ suggest that both low- and high-pressure discontinuities of ${T}_{c}$ have their origin in the Kohn anomalies are caused by the low-frequency phonons. However, details of the Fermi surface are different: the low-pressure anomaly is invisible in the band structure and it is associated with a global decrease of the nesting factor in the whole Brillouin Zone, while the high-pressure anomaly relates to a well-pronounced change in the band structure.

On the other hand, Tse et al.^7^ determined the Hall coefficient and its derivative as a function of the lattice constant by first-principles. Hall coefficient has slightly kinked at $6.1 {a}_{0}$ and $5.72 {a}_{0}$ (${a}_{0}$ is the lattice constant) corresponding to $5 \,{\text{GPa}}$ and $60 \,{\text{GPa}}$ pressure, respectively. The derivative at the same points shows appreciable discontinuity and slope variation in the electron–phonon coupling constant as a function of pressure, close to ${T}_{c}$^3^, so topological transitions are confirmed.

The absence of a precise theoretical explanation of the Nb ${T}_{c}$ anomalies measured reveals, one more time, the need for refinement of old and new models of the superconducting mechanism. A complete description of this behavior could lead us to new physics and a deeper understanding of the superconducting phenomenon.

In this paper, we report the Cooper Pairs Distribution functions ${D}_{cp}(\omega ,{T}_{c})$ for Nb (bcc) under pressure, which are built from the well-established Eliashberg spectral function ${\alpha }^{2}F\left(\omega \right)$ and phonon density of states (PhDOS), calculated by first-principles. These results allow an approach to the understanding of the Nb ${T}_{c}$ anomaly.

## Theory: Cooper Pairs Distribution function—${D}_{cp}(\omega ,{T}_{c})$

Conventional superconductivity is explained by an attractive electron interaction through lattice vibrations, which is possible under a set of specific physical conditions. We can associate a probability of occurrence for each of them^8–10^. Thus, simultaneous likelihood summed over all electronic states defines a distribution function that establishes the spectral range where Cooper pairs could be formed.

The population of vibrational states (bosons) at temperature $T$(${g}_{p}^{n}$), with energy between $\omega$ and $\omega +d\omega$, is determined by the density of vibrational states, ${N}_{ph}(\omega )$, times Bose–Einstein distribution at temperature $T$,

1 $${g}_{p}^{n}\left(\omega ,T\right)=\frac{{N}_{ph}\left(\omega \right)}{{e}^{\beta \omega }-1}$$

where $\beta ={k}_{B}T$ and ${k}_{B}$ is the Boltzman constant. The distribution for an additional state with energy $\omega$ at temperature $T$ is given by^11^,

2 $${g}_{p}^{n+1}\left(\omega ,T\right)={N}_{ph}\left(\omega \right)\left(1+\frac{1}{{e}^{\beta \omega }-1}\right)$$

For electrons (${g}_{e}^{n}$), the occupied states are described by electronic state density $N(\varepsilon )$, times Fermi factor at temperature $T$,

3 $${g}_{e}^{0}\left(\varepsilon ,T\right)=\frac{{N}_{e}\left(\varepsilon \right)}{{e}^{\left(\beta \omega -{E}_{F}\right)}+1}.$$

A corresponding distribution to vacant electronic states is given by,

4 $${g}_{e}^{v}\left(\varepsilon ,T\right)={N}_{e}\left(\varepsilon \right)\left(1-\frac{1}{{e}^{\left(\beta \omega -{E}_{F}\right)}+1}\right)$$

An electron and a phonon in a crystal may or may not interact between them. The electron–phonon coupling probability is associated with the Eliashberg spectral function ${\alpha }^{2}F(\omega )$. A measure of this probability is defined by the quotient ${\alpha }^{2}(\omega ) ={\alpha }^{2}F(\omega )/ {N}_{ph}(\omega )$. This quantity has been calculated for Nb at zero pressure by several authors^12,13^.

Now, the probability that a pair of electrons is coupled by a phonon with energy between $\omega$ and $\omega +d\omega$ is obtained by simultaneous likelihood, summed over all electronic states, that one electron with energy $\varepsilon$ interacts with the lattice and transfers to it an energy $\omega$, taking the $\varepsilon -\omega$ state, and a second electron with energy $\varepsilon ^{\prime}$, by interaction with the lattice, absorbs the phonon of energy $\omega$ and goes to ${\varepsilon }^{\prime}+\omega$ state, at the temperature ${T}_{c}$, ergo:

5 $${D}_{cp}\left(\omega ,{T}_{c}\right)={\int }_{{E}_{F}-{\omega }_{c}}^{{E}_{F}+{\omega }_{c}}{\int }_{{E}_{F}-{\omega }_{c}}^{{E}_{F}+{\omega }_{c}}{g}_{e}^{0}\left(\varepsilon ,{T}_{c}\right){g}_{e}^{v}\left(\varepsilon -\omega ,{T}_{c}\right){g}_{p}^{n+1}\left(\omega ,T\right){g}_{e}^{0}\left({\varepsilon }^{\prime},{T}_{c}\right){g}_{e}^{v}\left({\varepsilon }^{\prime}+\omega ,{T}_{c}\right){g}_{p}^{n}\left(\omega ,{T}_{c}\right){\alpha }^{2}\left(\omega \right)d\varepsilon d{\varepsilon }^{\prime}$$

where ${D}_{cp}\left(\omega ,{T}_{c}\right)$, namely Cooper Pairs Distribution function^9,10^, tells us the range of vibrational spectrum where the Cooper pairs are formed. Since electrons that form the Cooper pairs are near to the Fermi level ${E}_{F}$, interacting through the lattice phonons, it implies that these are into ${E}_{F}-{\omega }_{c}< \varepsilon < {E}_{F}+{\omega }_{c}$ energy interval. This cutoff phonon energy, ${\omega }_{c}$, should be the higher in the whole crystal-vibrational-spectrum $({\omega }_{max})$. However, for $T >0$ there are electrons occupying states with energy beyond Fermi level, then we could have $\varepsilon > {E}_{F}+{\omega }_{max}$ or $\varepsilon < {E}_{F}-{\omega }_{max}$. Accordingly, we choose ${\omega }_{c}$ such that to $\omega > {\omega }_{c}$, electronic energies do not have an appreciable contribution to ${D}_{cp}\left(\omega ,{T}_{c}\right)$.

Further, we can get an estimate of the total number of Cooper pairs formed at the temperature ${T}_{c}$ through a quantity proportional to it, the ${N}_{cp}$ parameter,

6 $${N}_{cp}={\int }_{0}^{{\omega }_{c}}{D}_{cp}\left(\omega ,{T}_{c}\right)d\omega $$

Next, we show the results of ${D}_{cp}\left(\omega ,{T}_{c}\right)$ and ${N}_{cp}$ evaluated at both pressure intervals that involve the anomalies at corresponding experimental ${T}_{c}$ values^3^.

## Results and discussion

**Figure 1:**
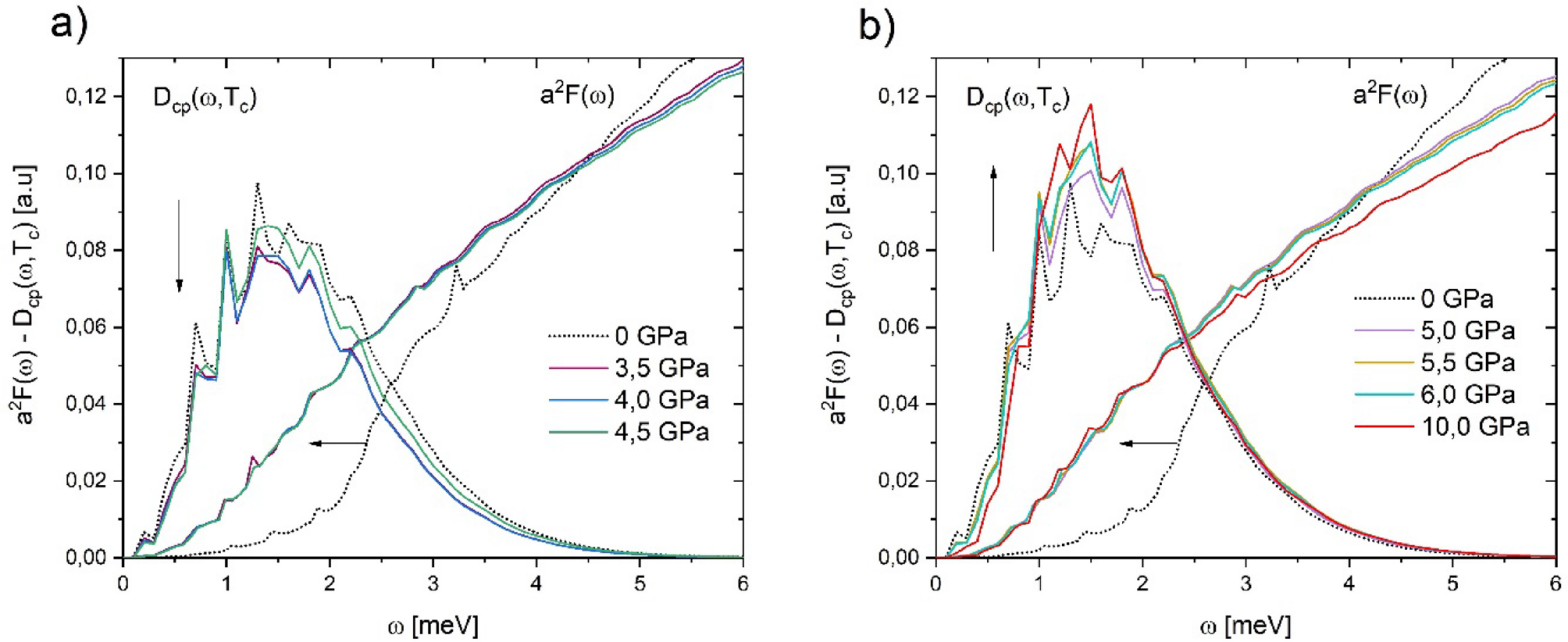
Cooper Pairs Distribution function ${D}_{cp}(\omega ,{T}_{c})$ and Eliashberg function for Niobium under pressure. (**a**) $0{-}4.5 \,{\text{GPa}}$ and (**b**) $5.0{-}10.0 \,{\text{GPa}}$. A detailed look of both (**a** and **b**) shows Eliashberg function smoothing respect to zero pressure, namely Nb anomaly, and their corresponding ${D}_{cp}(\omega ,{T}_{c})$ is located on this one.

**Figure 2:**
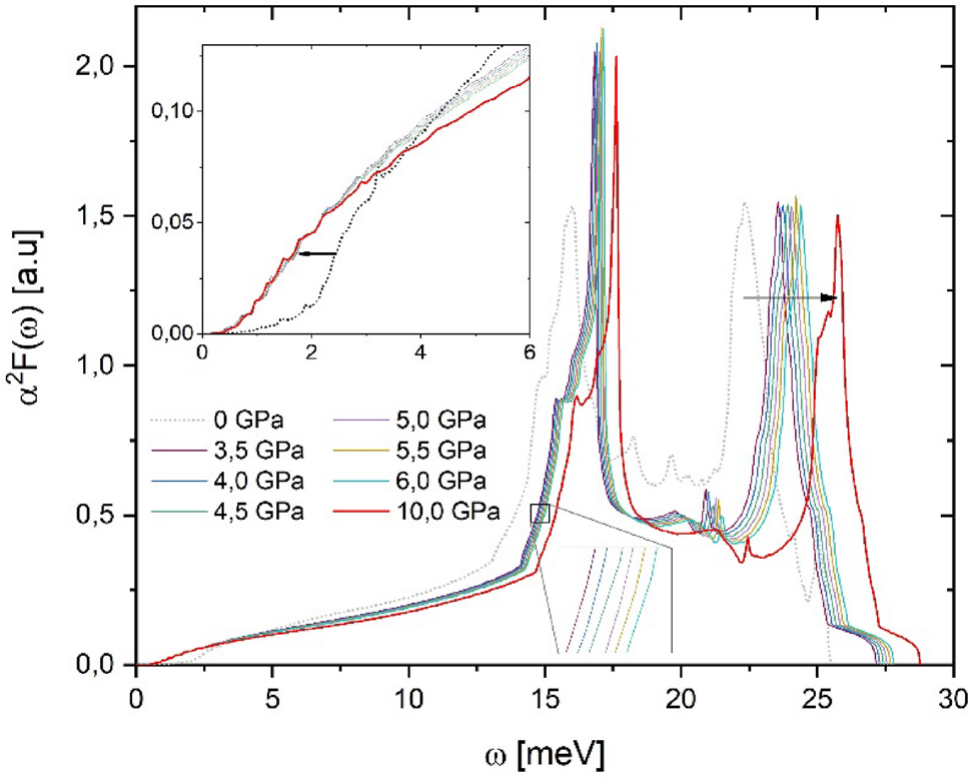
Eliashberg function $(0{-}10\,{\text{GPa}})$ for Niobium. Right arrow signs ${\alpha }^{2}F(\omega )$ hardening. Soothing interval of ${\alpha }^{2}F(\omega )$ (left arrow) is highlighted.

The Cooper Pairs Distribution functions ${D}_{cp}\left(\omega ,{T}_{c}\right)$ obtained for bcc Niobium under pressure (see Fig. 1) situates to Cooper-pairs formation in the 0–6 meV interval. It is observed that these ${D}_{cp}\left(\omega ,{T}_{c}\right)$ have a shape that mimics a Gaussian distribution centered around 1.5 meV. In general, the spectral Eliashberg function ${\alpha }^{2}F\left(\omega \right)$ moves towards high energies as pressure increases (see Fig. 2). However, in a narrow interval (0 to 4 meV) ${\alpha }^{2}F\left(\omega \right)$ spectra go back to low energy, as can be seen in Figs. 1 and 2. This behavior is in accordance with the work of Wierzbowska et al*.*^5,6^. By and large, our ${\alpha }^{2}F\left(\omega \right)$ spectra are in good agreement with previous theoretical reports^12–14^.

At the range pressures of $0{-}10\,{\text{GPa}}$, the superconducting critical temperature Niobium undergoes a decrease with an ulterior raising^3^, namely Nb anomaly. The tendency change happens at $4.0 \,{\text{GPa}}$, approximately^3,15^. Nevertheless, electronic band structure and Fermi surface do not suggest any evident relationship with the Nb anomaly^4,16–24^. When we look at Eliashberg functions (Fig. 2), we do not find a visible variation that we can associate with the Nb anomaly, except for the narrow interval from 0 to 4 meV. On a first view, this interval has a subtle or no contribution to Nb superconductivity. But this interval claims its importance in superconducting properties when is observed that Cooper pair formation (around 1.5 meV) coincides with this one (see Fig. 1).

Theoretical and experimental studies about Nb Fermi surface reported by several authors^4,16–23^ do not detect any appreciable change at low pressure. However, Wierzbowska et al*.*^5,6^, by nesting factor, found slight features of the Nb Fermi surface. This could be associate with the Nb anomaly. On the other hand, Struzhkin et al*.*^3^ and one previous study^9^ showed that the electron–phonon parameter $\lambda$, has a good correlation with the critical temperature behavior. However, ours ${D}_{cp}\left(\omega ,{T}_{c}\right)$ specifically show that a small region of the whole vibrational spectrum contributes to the Cooper pair formation (Fig. 1).

The ${D}_{cp}\left(\omega ,{T}_{c}\right)$ at pressures $0\le P\le 10 \,{\text{GPa}}$, are located around 1.5 meV, precisely in the spectral range where the corresponding ${\alpha }^{2}F\left(\omega \right)$ undergoes softening. Then, we could associate this demeanor with the Nb anomaly. Further, ${D}_{cp}\left(\omega ,{T}_{c}\right)$ marks the change of the tendency of ${T}_{c}\left(P\right)$ around $4.0 \,{\text{GPa}}$. The ${D}_{cp}\left(\omega ,{T}_{c}\right)$ at $0<P\le 4.5 \,{\text{GPa}}$ are always under ${D}_{cp}\left(\omega ,{T}_{c}\right)$ calculated at zero pressure (see the arrow down in Fig. 1a), while at $4.5<P\le 10 \,{\text{GPa}}$ ${D}_{cp}\left(\omega ,{T}_{c}\right)$ are over it (see arrow up in Fig. 1b). This behavior allows us to infer that there is a change in physical conditions at ~ $4.0 \,{\text{GPa}}$, which could be inducing the Nb anomaly.

**Figure 3:**
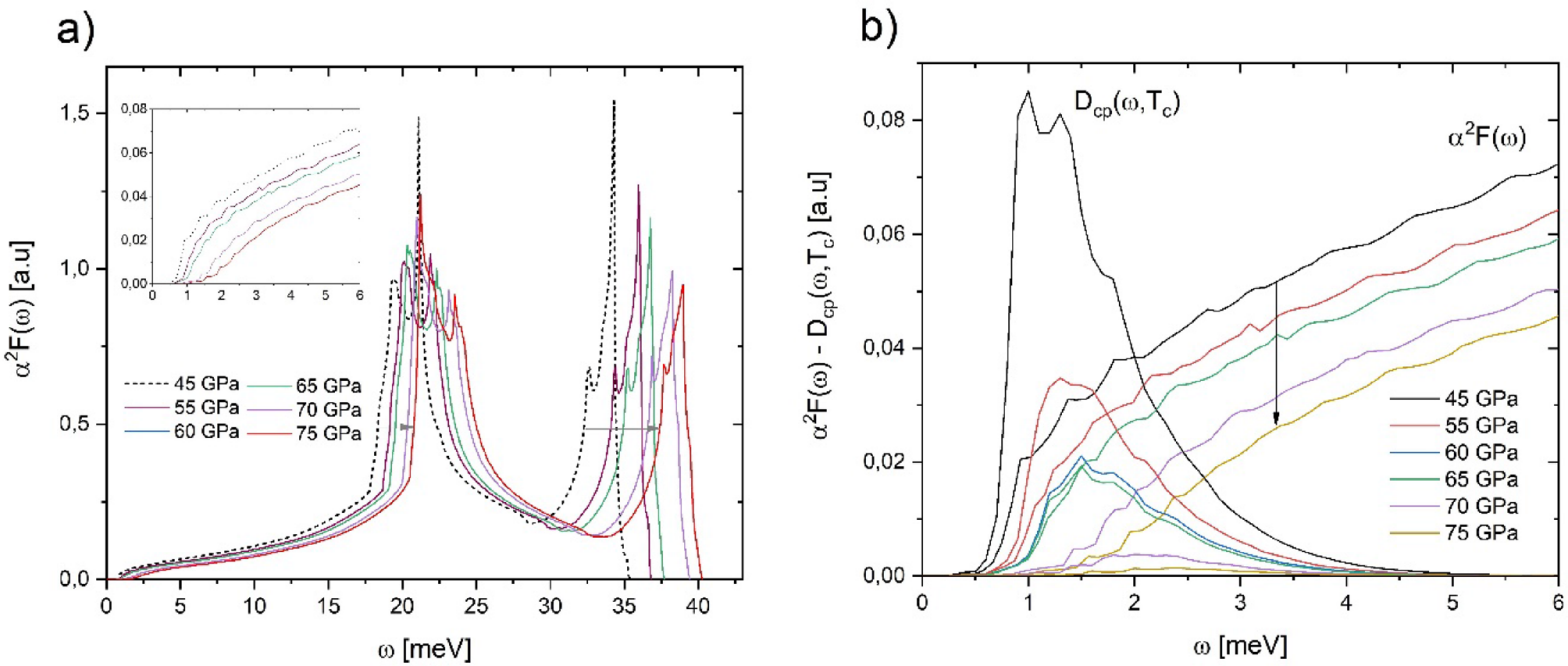
(**a**) Eliashberg function ${\alpha }^{2}F(\omega )$ and (**b**) Cooper Pairs Distribution function ${D}_{cp}(\omega ,{T}_{c})$ for Niobium at $45{-}75\,{\text{GPa}}$ pressures.

On the other hand, the second Nb anomaly occurs around $50 \,{\text{GPa}}$, where ${T}_{c}$ begins to decrease significantly^3^. This behavior has been explained by the electronic band structure and Fermi level variations and the trend of weakening electron–phonon interaction under pressure^3,4,7^. Our results show that Eliashberg functions harden at the pressure range of $45{-}75\,{\text{GPa}}$ (Fig. 3a). A detailed view of ${\alpha }^{2}F(\omega )$ at low energies (Fig. 3b) displays a clear diminution of the electron–phonon interaction. In this case, the pressure effects are more evident than at low pressures.

All ${D}_{cp}\left(\omega ,{T}_{c}\right)$ calculated at the pressure range of $45{-}75\,{\text{GPa}}$ reveal a Gaussian shape and are in the 0–5 meV interval (Fig. 3b). A significant decrease in ${D}_{cp}\left(\omega ,{T}_{c}\right)$ with increasing pressure is observed, mainly between 45 and 55 GPa where the Nb anomaly has been measured. Due to ${\alpha }^{2}F(\omega )$ decreasing as pressure increases (at low energies), the Cooper pairs formation falls, and in that way, ${T}_{c}$ does too. So, as at low pressure $(0{-}10\,{\text{GPa}})$, the low energy phonons take remarkable importance in the behavior of ${T}_{c}$.

**Figure 4:**
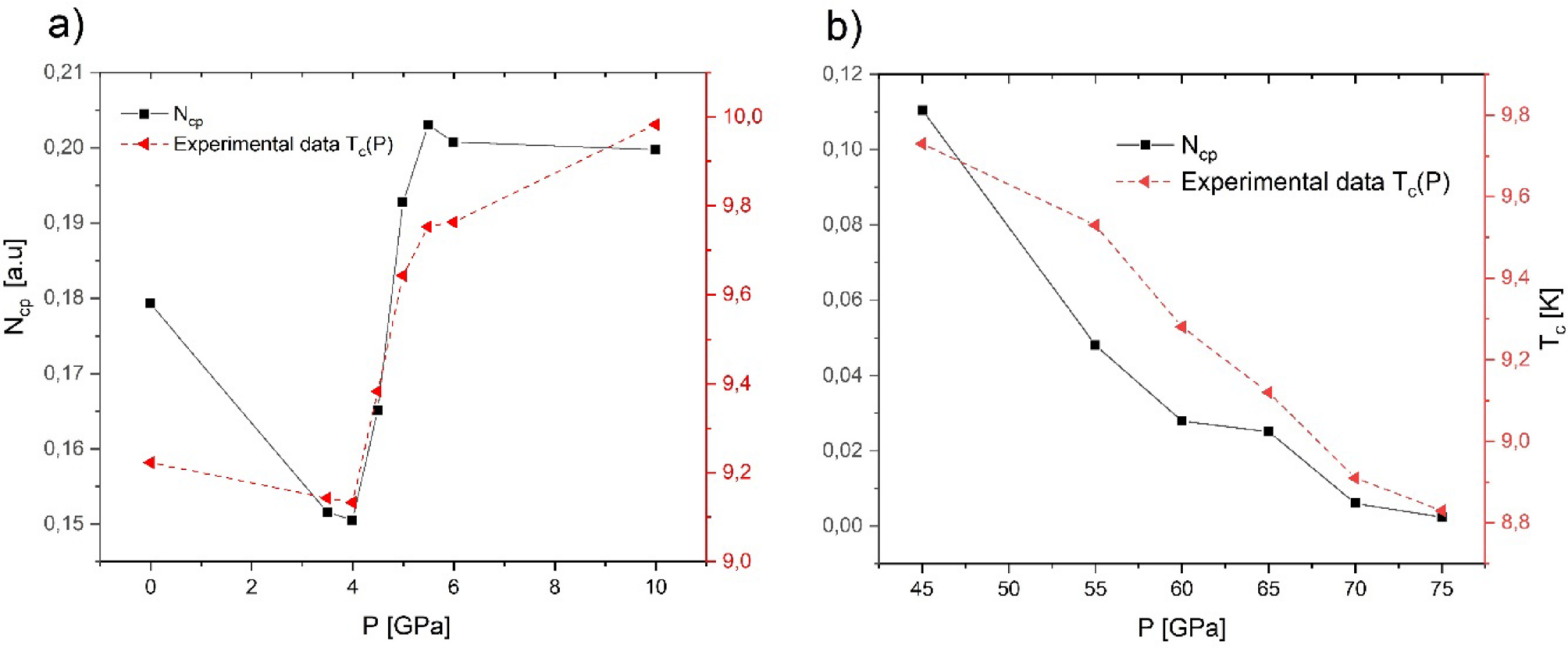
${T}_{c}$ experimental data^3^ and ${N}_{cp}$ as a function of pressure for Nb: (**a**) $0\le P\le 10 \,{\text{GPa}}$ and (**b**) $45\le P\le 75 \,{\text{GPa}}$.

From ${D}_{cp}(\omega ,{T}_{c})$, it was possible to obtain the ${N}_{cp}$ parameter, which is proportional to the total number of Cooper-Pairs formed at a temperature ${T}_{c}$. We found that the ${N}_{cp}$ parameters calculated (for both pressure intervals) have a significant correlation with experimental data for ${T}_{c}$. It is observed that ${N}_{cp}$ as a function of pressure reproduce well the trend of ${T}_{c}(P)$ measured (see Fig. 4).

According to our results (Fig. 4), we can infer that the pressure induces modifications on the physical conditions (at low-energies) that lead to generation (or reduction) of the number of Cooper pairs, which cause the increase (or decrease) of the ${T}_{c}$.

Finally, the analysis from ${D}_{cp}\left(\omega ,{T}_{c}\right)$ bring us to suggest an experiment where low-energy phonon could be stimulated in superconducting samples to research ${T}_{c}$ improvement. These results validate the use of ${D}_{cp}(\omega ,{T}_{c})$ as a theoretical tool for the study of conventional superconductors.

## Conclusions

Here, we presented the Cooper Pair Distribution functions ${D}_{cp}(\omega ,{T}_{c})$ for Nb (bcc), calculated by first-principles. These results enabled us to broaden the understanding of the anomalous ${T}_{c}(P)$ behavior measured in the Nb, in the pressure ranges of $0{-}10\,{\text{GPa}}$ and $45{-}75\,{\text{GPa}}$. ${D}_{cp}(\omega ,{T}_{c})$ showed that the Cooper-pairs formation energy intervals are located at low-energies $(\omega <6 \,{\text{meV}})$. At low-pressures $(P < 10 \,{\text{GPa}})$, the low-energy region of ${\alpha }^{2} F(\omega )$ moves towards lower frequencies in the same region of the Cooper pairs formation energy interval, which leads to the recognition of the importance of low-energy phonons in the superconducting behavior of Nb under pressure, despite their slight spectral weight. From ${D}_{cp}(\omega ,{T}_{c})$ it was possible to obtain the ${N}_{cp}$ parameter, which is proportional to the total number of Cooper pairs formed at different ${T}_{c}$ and pressures. ${N}_{cp}$ as a function of pressure achieved an adequate reproduction of the trend of ${T}_{c}(P)$ measured, that is to say, the Nb anomalies around 5 and 50 GPa. From our ${D}_{cp}(\omega ,{T}_{c})$ findings, it is expected that electron–phonon interaction specifically at low-energies $(\omega <6\,{\text{meV}})$ contributes to superconducting properties, despite the ${\alpha }^{2} F(\omega )$ having a stronger spectral value at high vibrational energy. All of our results validate the use of ${D}_{cp}(\omega ,{T}_{c})$ as a theoretical tool for the study of conventional superconductors.

## Methods

In order to determine the Cooper Pair Distribution function ${D}_{cp}(\omega ,{T}_{c})$, we require electronic density states, vibrational density states, and Eliashberg function. To do these ab initio calculations, we first relax the internal degrees of freedom and the lattice vectors of the Nb structure using the Broyden–Fletcher–Goldfarb–Shanno (BFGS) quasi-Newton algorithm^25–28^ at each pressure to get the corresponding lattice constants. From these relaxed structure configurations, we calculated the electronic and phonon band structures, electron (DOS) and phonon (PHDOS) densities of states, and the Eliashberg function ${\alpha }^{2}F(\omega )$. We used a kinetic energy cut-off of $80 Ry$ for the expansion of the wave function into plane waves and $320 Ry$ for the density. To integrate over the Brillouin zone (BZ), we used for the electronic integration a $k$-grid of $32\times 32\times 32$ and for the phononic integration a $q$-grid of $8\times 8\times 8$ according to the Monkhorst–Pack scheme^29^. The calculations were done with the pseudopotential plane-wave (PW) method of Perdew et al*.*^30^, using the generalized gradient approximation (GGA) and the Troullier and Martins norm-conserving pseudopotential^31^. The cut-off and grids were chosen big enough as to obtain a good precision in ${\alpha }^{2}F(\omega )$ calculated within the density-functional perturbation theory (DFPT) frame^32,33^. We used the Quantum Espresso code^34^ for all these calculations.
